# Health utility assessments in individuals undergoing diagnostic and surveillance colonoscopy: improved discrimination with a cancer-specific scale

**DOI:** 10.1007/s10552-023-01789-6

**Published:** 2023-09-25

**Authors:** Norma B. Bulamu, Gang Chen, Ellen McGrane, Charles Cock, Graeme P. Young, Erin L. Symonds

**Affiliations:** 1 Flinders Health and Medical Research Institute, College of Medicine and Public Health, Flinders University, Adelaide, South Australia Australia; 2 Monash Business School, Centre for Health Economics, Monash University, Melbourne, VIC Australia; 3 School of Health and Related Research (ScHARR), The University of Sheffield, Sheffield, UK; 4 Gastroenterology Department, Flinders Medical Centre, Adelaide, South Australia Australia

**Keywords:** EQ-5D-5L, QLU-C10D, Colorectal cancer, Discriminant validity, Health-related quality of life

## Abstract

**Purpose:**

To compare the sensitivity and discriminant validity of generic and cancer-specific measures for assessing health-related quality of life (HRQoL) for individuals undergoing diagnostic or surveillance colonoscopy for colorectal cancer.

**Methods:**

HRQoL was assessed using EQ-5D-5L (generic), and EORTC QLQ-C30 (cancer-specific) scales, 14 days after (baseline) and one-year following colonoscopy (follow-up). Utility scores were calculated by mapping EORTC-QLQ-C30 onto QLU-C10D. Differences between participants with different indications for colonoscopy (positive faecal occult blood test (FOBT), surveillance, or symptoms) and colonoscopy findings (no polyps, polyps, or cancer) were tested using Wilcoxon-Mann–Whitney and Kruskal–Wallis H tests. Sensitivity was assessed by calculating the ceiling effects (proportion reporting the best possible level).

**Results:**

246 adults completed the survey, including those undergoing colonoscopy for symptoms (*n* = 87), positive FOBT (*n* = 92) or surveillance (*n* = 67). Those with symptoms had the lowest HRQoL at both baseline and follow-up, with differences observed within the HRQoL domains/areas of role function, appetite loss and bowel function on the QLU-C10D. No differences were found in HRQoL when stratified by findings at colonoscopy with both measures or when comparing baseline and follow-up responses. Participants reporting full health with EQ-5D-5L (21% at baseline and 16% at follow-up) still had problems on the QLU-C10D, with fatigue and sleep at baseline and with role function and fatigue at follow-up.

**Conclusion:**

Patients undergoing colonoscopy for symptoms had lower HRQoL compared to surveillance or positive FOBT. The cancer-specific QLU-C10D was more sensitive and had greater discriminant ability between patients undergoing colonoscopy for different indications.

**Supplementary Information:**

The online version contains supplementary material available at 10.1007/s10552-023-01789-6.

## Introduction

Colorectal cancer is the third most diagnosed cancer and is responsible for 11% of cancer deaths in Australia [1], and other developed countries [2, 3]. Colorectal cancer (CRC) has a five-year survival of around 70% which is mainly attributable to finding cancer in its early stages [4, 5]. This is achieved with screening programs, such as with colonoscopy, or through utilising faecal occult blood tests (FOBT) followed by diagnostic colonoscopy for those returning a positive screening test result [6–8]. In addition, regular surveillance colonoscopy for individuals deemed at elevated risk (those with a previous neoplastic lesion or a significant family history of colorectal cancer) reduces the incidence of and mortality of CRC [9, 10]. People at elevated risk for CRC are generally recommended to undergo colonoscopy every three to five years [11]. With the increasing number of colonoscopy procedures worldwide, attention must be paid to the delivery of care, which can be informed by the assessment of patient-reported outcomes.

Screening and surveillance colonoscopy reduces mortality and the incidence of CRC through adenoma removal [12–14]; however, the colonoscopy procedure is associated with discomfort, pain and a risk of adverse events such as perforation [15]. It is also argued that knowing one’s results after a colonoscopy may be associated with a certain degree of anxiety depending on the nature of the results [16–18]. As such, both the procedure and diagnostic results may have an impact on health-related quality of life (HRQoL). Several of these studies reporting the impact of CRC screening on patient-reported outcomes such as HRQoL have applied generic measures such as the SF-36 [18], or non-validated scales specifically designed for the studies [16]. Studies assessing HRQoL following a diagnosis of cancer show that generic measures may not be sensitive to changes in HRQoL outcomes in these populations [19–21]. Yet while the number of cancer-specific measures and studies applying these tools/measures in individuals with cancer has increased [22, 23], there is a paucity of research investigating their use to assess changes in HRQoL in people undergoing diagnostic (following a positive FOBT or symptoms) and surveillance colonoscopy for CRC, as well as limited studies comparing them to generic measures.

This study, therefore, assessed HRQoL for individuals undergoing diagnostic or surveillance colonoscopy for CRC, using both generic and cancer-specific measures. The aim was to assess the sensitivity and discriminant validity of two multi-attribute utility measures (MAUI), the generic EQ-5D-5L and cancer-specific EORTC Quality of Life Utility Measure-Core 10 dimensions (QLU-C10D) for individuals undergoing colonoscopy for different indications related to CRC detection. Multi-attribute utility measures are used to assess the quality of life and generate utility estimates for the calculation of quality-adjusted life years (QALYs), the outcome measure required for cost-utility analysis (CUA). With the increasing use of CUA in the assessment of health interventions, it is important to determine the appropriate instrument/scale for a given population to inform the accuracy of utility and cost-effectiveness results.

## Methods

### Study population

This was a prospective study of an Australian population who had recently undergone a colonoscopy in a public hospital setting (Flinders Medical Centre or Noarlunga Hospital, South Australia). We reviewed clinical records to identify patients aged ≥ 40 years who had a recent colonoscopy and invited them into the study. Individuals were excluded if they had prior treatment for CRC or had a pre-existing and ongoing bowel condition that required medication or was the indication for the colonoscopy (such as inflammatory bowel disease).

The survey was mailed out approximately 14 days after the colonoscopy. This included study information, a consent form and the HRQoL scales, first the generic EQ-5D-5L followed by the cancer-specific scale. For participants who responded to the first survey, a repeat survey was sent one year later. A reminder phone call was made if the survey had not been completed and returned within two weeks.

### Clinical measures

All study invitees underwent either a diagnostic colonoscopy to investigate the cause of symptoms, a follow-up after a positive FOBT screening test, or a surveillance colonoscopy due to an elevated risk for CRC [10, 11]. Colonoscopy findings were reviewed, and diagnosis was classified based on whether any type of polyp was removed, or whether colorectal cancer was diagnosed (divided into early stage (I and II) and advanced stage (III and IV)). Polyps were not divided into subclasses (e.g., advanced or non-advanced adenomas, sessile-serrated lesions, benign polyps) as it was felt that this level of discrimination would not be appropriate for most individuals. Patients’ pathology knowledge is often limited to whether anything was found and removed at colonoscopy, and whether it was cancer [24, 25].

### Health-related quality of life

The survey collected information on participant demographics (including age, gender, marital status, socioeconomic status (based on the Socio-Economic Indexes for Areas (SEIFA) score), work status, having private health insurance and education level), health status (including having a disability, comorbidities and previous history of surgery and cancer) and HRQoL assessment.

HRQoL was assessed using the EQ-5D-5L [26], which is a generic multi-attribute utility instrument and the cancer-specific EORTC QLQ-C30 [27]. The use of a cancer-specific scale was considered appropriate in this population (both diagnostic and surveillance colonoscopies) as studies have shown that these individuals can be fearful of a cancer diagnosis [28–30].

The generic EQ-5D-5L has five dimensions: mobility, self-care, usual activities, pain/discomfort and anxiety/depression with responses across five levels; no problems, slight problems, moderate problems, severe problems and extreme problems [26]. As recommended by the UK National Institute of Care Excellence (NICE) utility scores for EQ-5D-5L were generated using the EQ-5D-5L crosswalk tariff developed from a general population sample in the UK [31].

The cancer-specific EORTC-QLQ-C30 has one global HRQoL scale, five functional scales (physical, role, emotional, cognitive, social), three symptom scales (fatigue, nausea or vomiting, pain) and six single items (sleeping disorders, appetite loss, dyspnoea, diarrhoea, constipation and financial problems). Each item has four alternative responses (1—not at all; 2—a little; 3—quite a bit; 4—very much) [27]. The responses were mapped onto the QLU-C10D, a utility scoring algorithm developed by King et al. to generate utility scores [32]. The QLU-C10D has four functional scales and six symptom scales, each with four levels: not at all, a little, quite a bit, and very much. The functional scales are physical function, role function, social function and emotional functioning while the symptom scales are pain, fatigue, sleep, appetite, nausea and bowel problems. The value set for the QLU-C10D was based on an Australian general population sample with theoretical utility scores ranging from -0.095 to 1 [33].

### Data analysis

Data were analysed using Stata version 15 (StataCorp, College Station, TX, USA). Participant characteristics were summarised as means and standard deviations (SD) for continuous variables and absolute numbers and percentages for categorical variables.

#### Health-related quality of life

Descriptive statistics including means, medians and ranges were compared for each instrument at baseline (immediately after colonoscopy) and during follow-up (one year after colonoscopy). Differences in HRQoL between the two time points were explored using Wilcoxon–Mann–Whitney sign rank test.

#### Instrument sensitivity

Lower ceiling effects suggest greater sensitivity and discriminant ability of an instrument. The ceiling effect occurs when the highest possible level of a dimension or score of an instrument or measure is achieved in more than 15% of respondents [34]. The ceiling effect for EQ-5D-5L was calculated as the proportion of ‘no problem’ responses in each dimension and the proportion of ‘no problem’ in all dimensions. QLU-C10D ceiling effects were calculated as the proportion of level 1 (highest level) on each dimension and all dimensions. Ceiling effects were further explored by examining those reporting full health in one instrument to assess what was reported in the other instrument.

#### Discriminant validity

Discriminant validity is an instrument's ability to measure expected differences between subgroups of patients [35, 36]. Means and SDs were compared between the different indications for and diagnoses at colonoscopy. The indications categories were surveillance, positive FOBT and symptoms. For the diagnoses, comparisons were made in those with and without polypectomy; those with and without cancer; and those with advanced cancer (stages III and IV) compared to those with no cancer or less advanced stages of cancer (stages I & II). Wilcoxon–Mann–Whitney test and the Kruskal–Wallis H test for non-normally distributed data were used to test for differences between subgroups. Differences between subgroups were also explored at the dimension level for both EQ-5D-5L and QLU-C10D.

The discriminant abilities of both EQ-5D-5L and QLU-C10D were further explored using Tobit regression models, with adjustment for potential confounders of HRQoL to reduce bias [37]. Confounders considered included age, gender, marital status, having a disability and comorbidities which are known to affect health-related quality of life [38–40]. In addition, employment, having private health insurance and education level as proxies for socioeconomic status [41] and previous history of cancer, history of surgery as well the indication for colonoscopy were considered as proxies for baseline health status [40]. Univariate analysis using spearman correlation was undertaken, and only variables with a significant correlation to HRQoL were included in the final regression model.

Tobit regression was applied because the HRQoL data were skewed with over 20% of respondents reporting full health at both baseline and follow-up for EQ-5D-5L and 10% for QLU-C10D. The best-fitting model was determined based on the log-likelihood, and a p-value of 0.05 was considered statistically significant.

## Results

### Study sample

The survey was sent to 644 individuals who had undergone colonoscopies between March 2017 and July 2019. Demographic details of participants are provided in Table 1. The flow chart in Figure S1 shows the categories of participants.

**Table 1:** Demographic characteristics for participants of each survey

Variable	Baseline (*N* = 246)		Follow-up (*N* = 176)		*p* value	
Male	134 (54%)		95 (54%)		0.50	
Low socioeconomic status (based on SEIFA scores)*	109 (44%)		71 (41%)		0.23^2^	
Married	156 (63%)		108 (61%)		** < 0.001**^**b**^	
Higher Education^a^	113 (46%)		83 (47%)		**0.001**^**b**^	
Full Time Worker	54 (22%)		33 (19%)		**0.01**^**b**^	
Retired	108 (44%)		83 (33.3%)		**0.01**^**b**^	
Have private health insurance	99 (40%)		74 (43%)		0.65^b^	
Have a disability	57 (23%)		37 (21%)		0.31^b^	
Had surgery in the last 12 months	63 (27%)		43 (26%)		0.30^b^	
Previous or current cancer	61 (25%)		`45 (26%)		0.55^b^	
Age [mean (SD)]	64.2 (8.2)		65.2 (7.7)		** < 0.001**^**c**^	
Age category						
< 55	33 (14%)		13 (7%)			
55–65	94 (39%)		68 (39%)			
66–75	103 (43%)		84 (48%)			
> 75	12 (5%)		9 (5%)			
HRQoL survey completion						
Days after colonoscopy results Median (IQR)	38 (34, 43)		423 (414, 437)		N/A	
Indication for colonoscopy						
Surveillance	67 (27%)		54 (32%)			
Positive FOBT	92 (37%)		65 (37%)			
Symptoms	87 (35%)		57 (31%)			
Colonoscopy findings						
No polyp	75 (30%)		57 (32%)			
Polyp	128 (50%)		91 (52%)			
Cancer	44 (17%)		24 (14%)			
Stage I and stage II	22		13			
Stage III and stage IV	22		11			
Colonoscopy findings for different Indications for colonoscopy at baseline and follow-up						
Indication	No polyps		Polyps		Cancer	
Surveillance	20 (31%)	16 (30%)	43 (66%)	36 (68%)	2 (3%)	1 (2%)
Positive FOBT	23 (26%)	20 (31%)	51 (57%)	36 (56%)	15 (17%)	8 (13%)
Symptoms	28 (33%)	21 (38%)	31 (36%)	19 (35%)	26 (31%)	15 (27%)

^a^Education level above Grade 12 – technical certificate, diploma and degrees

^b^Two sample test of proportions- difference between baseline and follow-u

^c^Wilcoxon signed ranksum test

^*^SEIFA Socio-economic indexes for areas

246 respondents completed the surveys at baseline, a median of 38 days (IQR: 34, 43) after colonoscopy and 176 at follow-up, a median of 423 days (IQR: 414, 437) after colonoscopy. The baseline sample was predominantly male (54%) with a mean age of 64 years (SD = 8.2) and did not have private health insurance (60%). Slightly more respondents had a diagnostic colonoscopy because of a positive FOBT (37%) or symptoms (36%) compared to surveillance (26%), and 50% of the cohort underwent polypectomy at colonoscopy (Table 1). Sixty-nine (69) respondents to the baseline survey did not return the follow-up survey (Table S1). Non-responder demographic characteristics were similar to responders with differences observed in the indication for colonoscopy (46% undertaking a colonoscopy due to symptoms compared to 31% responders) and colonoscopy findings (29% diagnosed with cancer compared to 14% responders).

### Health-related quality of life

HRQoL for the whole cohort did not differ between baseline and follow-up for both EQ-5D-5L [0.76 (SD = 0.22) and 0.76 (SD = 0.20), *p value* = *0.23*] and QLU-C10D [0.74 (SD = 0.21 and 0.76 (SD = 0.2), *p value* = *0.58*]. Marginally higher scores were observed with EQ-5D-5L than QLU-C10D at baseline, but scores were the same at follow-up.

### Ceiling effects of EQ-5D-5L and QLU-C10D

24% (60/246) of respondents reported having the best possible level (no problems) for all dimensions of EQ-5D-5L at baseline and 22% (38/176) at follow-up, while 4% (11/246) and 6% (11/176) had the best possible levels, respectively, for QLU-C10D (Figure S2 and S3). Over 15% of respondents reported the highest level with all dimensions of EQ-5D-5L and QLU-C10D at both baseline and follow-up, which was an indication of ceiling effects [42].

**Fig. 1:**
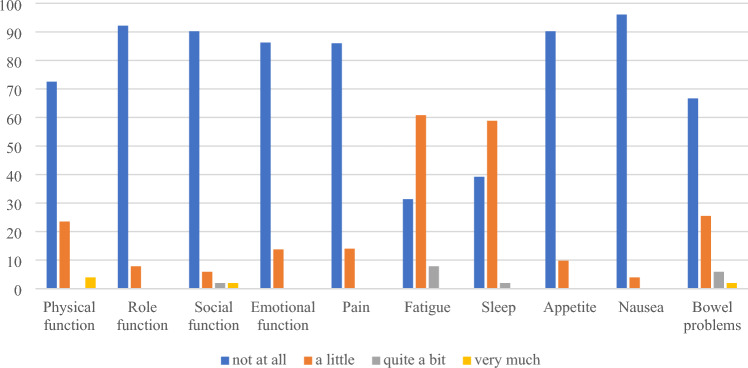
Distribution (%) of QLU-C10D dimension responses for participants with full health on EQ-5D-5L but not on QLU-C10D at baseline (*n* = 51)

**Fig. 2:**
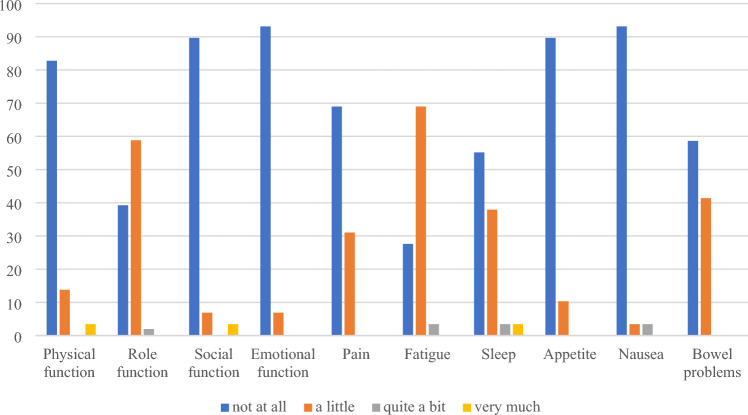
Distribution (%) of QLU-C10D dimension responses for participants with full health on EQ-5D-5L but not on QLU-C10D at follow-up (*n* = 29)

51 (21%) respondents at baseline and 29 (16%) at follow-up reported full health (utility score = 1) on EQ-5D-5L but not on QLU-C10D. Participants reporting full health with EQ-5D-5L (no problems for all dimensions) still had problems with QLU-C10D, particularly with fatigue (61%) and sleep (59%) at baseline, and with role function (59%) and fatigue (69%) at follow-up where the majority reported less than the best possible level on QLU-C10D (Fig. 1 and 2 and Table S2).

No floor effects were observed with under 2.5% of respondents reporting the lowest levels on each dimension of the EQ-5D-5L and under 10% with QLU-C10D except for physical functioning where 33.5% reported the lowest level at follow-up (see figure S4 and S5).

### Discriminant validity of EQ-5D-5L and QLU-C10D – bivariate analysis

Table 2 shows the ability of both measures to discriminate between participants with different colonoscopy findings and indications for colonoscopy. Neither measure discriminated between participants with different colonoscopy findings at both time points but discriminated between different indications for colonoscopy. Participants receiving colonoscopy because of symptoms had lower HRQoL at both baseline and follow-up as assessed by both EQ-5D-5L 0.71 (0.21), *p value* = *0.001* and 0.72 (0.20) *p-value* = *0.015*) and QLU-C10D 0.67 (0.21), *p value* =  < *0.001* and 0.67 (0.21), *p value* =  < *0.001*).

**Table 2:** Discriminant validity of HRQoL measures between colonoscopy findings and indications for colonoscopy indications—Bivariate analysis

	Baseline		Follow-up	
EQ-5D-5L				
Colonoscopy finding	Mean (SD)	*p* value^a^	Mean (SD)	*p* value^a^
No Polyp	0.78 (0.20), [*n* = 70]	0.38	0.77 (0.18), [*n* = 57]	0.90
Polyp	0.75 (0.22), [*n* = 124]		0.76 (0.19), [*n* = 91]	
Non-Cancer^b^	0.76 (0.22), [*n* = 201]	0.55	0.77 (0.19), [*n* = 152]	0.76
Cancer^c^	0.78 (0.23), [*n* = 44]		0.74 (0.26), [*n* = 24]	
Non-Cancer and Early-Stage Cancer^d^	0.76 (0.22), [*n* = 223]	0.78	0.77 (0.21), [*n* = 165]	0.32
Advanced Stage Cancer^e^	0.76 (0.19), [*n* = 22]		0.75 (0.09), [*n* = 11]	

Indication for colonoscopy				
	Mean (sd)	*p*-value^f^	Mean (sd)	*p*-value^f^
Surveillance	0.78 (0.21), [*n* = 65]	***0.0005***	0.78 (0.19), [*n* = 54]	***0.015***
Positive FOBT	0.80 (0.22), [*n* = 92]		0.80 (0.20), [*n* = 65]	
Symptoms	0.71 (0.21), [*n* = 88]		0.72 (0.20), [*n* = 57]	

QLU-C10D				
Colonoscopy finding	Mean (sd)	*p*-value^b^	Mean (sd)	*p*-value^b^
No Polyp	0.76 (0.20), [*n* = 71]	0.72	0.76 (0.20), [*n* = 57]	0.58
Polyp	0.74 (0.21), [*n* = 125]		0.78 (0.19), [*n* = 91]	
Non-Cancer^b^	0.74 (0.21), [*n* = 203]	0.28	0.77 (0.19), [*n* = 152]	0.13
Cancer^c^	0.71 (0.22), [*n* = 43]		0.70 (0.21), [*n* = 23]	
Non-Cancer and Early-Stage Cancer^d^	0.74 (0.21), [*n* = 225]	0.11	0.76 (0.20), [*n* = 164]	0.08
Advanced Stage Cancer^e^	0.70 (0.16), [*n* = 21]		0.70 (0.15), [*n* = 11]	

Indication for colonoscopy				
	Mean (sd)	*p*-value^f^	Mean (sd)	*p*-value^f^
Surveillance	0.77 (0.20), [*n* = 67]	0.0001*	0.81 (0.18), [*n* = 55]	0.0002*
Positive FOBT	0.77 (0.21), [*n* = 92]		0.79 (0.17), [*n* = 65]	
Symptoms	0.67 (0.21), [*n* = 87]		0.67 (0.21), [*n* = 55]	

^a^Wilcoxon Mann–Whitney *U* test used to test differences between Non-Cancer and Cancer, and between Non advanced and Advanced cancer

^b^Non-cancer = normal, non-neoplastic and polyp

^c^Cancer = all stages of cancer

^d^Early stage cancer = cancer stage I and II

^e^Advanced cancer stage = cancer stage III and IV

^f^kwallis test for difference between groups *Statistically significant at p-value

### Responses to dimensions of the EQ-5D-5L at baseline

At the dimension level (Figure S6), significant differences between indications for colonoscopy were observed in individual responses to EQ-5D-5L dimensions of usual activities (*p value* = *0.04*), pain/discomfort (*p value* = *0.03*), and anxiety/depression (*p value* = *0.05*) at baseline (Fig. 3). Significantly less symptomatic individuals reported the best two levels [no problems or slight problems] for usual activities and pain/discomfort compared to individuals undergoing colonoscopy for surveillance or positive FOBT. However, more participants undergoing colonoscopy because of positive FOBT or symptoms reported the best two levels for anxiety/depression compared to those under surveillance.

### Responses to dimensions of the QLU-C10D at baseline

Significant differences were observed at the dimension level with the QLU-C10D between the different colonoscopy findings (Figure S7) and indications for colonoscopy (Figure S8). More participants with no polyps compared to those having polyps reported no trouble or a little trouble (best two levels) with physical functioning (*p value* = *0.05*) and pain (*p value* = *0.04*). Differences in the appetite dimension were observed between respondents with no cancer and those with a cancer diagnosis (*p value* = *0.003*) as well as no cancer or early-stage cancer and advanced cancer (*p value* = *0.002*). More participants with no cancer or early-stage cancer reported the best level of appetite compared to cancer and advanced cancer, respectively.

Figure S6 shows the significant differences with QLU-C10D at baseline observed between indications for colonoscopy with role function (*p value* = *0.01*), appetite (*p value* = *0.01*), and bowel problems (*p value* = *0.01*), with significantly more participants undergoing colonoscopy for symptoms reporting the lower two levels (quite a bit of trouble and very much trouble) and less reporting the higher two levels (not at all and a little trouble) than those having a colonoscopy for surveillance or positive FOBT.

### Responses to dimensions of the EQ-5D-5L at follow-up

No significant differences between colonoscopy findings or indications for colonoscopy were observed in individual responses to EQ-5D-5L dimensions at follow-up.

### Responses to dimensions of the QLU-C10D at follow-up

Significant differences were observed between participants with no cancer and cancer (all stages) in responses to dimensions of physical functioning (*p value* = *0.02*) and social functioning (*p value* = *0.03*) as well as those with early-stage cancer and advanced cancer (*p value* = *0.04* and *p value* = *0.01*). More participants with cancer and advanced cancer reported high levels of trouble with physical functioning and social functioning compared to those without cancer and with no cancer or early-stage cancer (Figure S9). With the symptom scales, a significant difference was only observed with appetite (*p value* = *0.03*) when the severity of cancer was considered where only 45% of respondents with advanced cancer reported no lack of appetite compared to 81% of those with no cancer or early-stage cancer (Figure S9).

Consistent with findings at baseline, more participants undergoing colonoscopy due to symptoms had trouble with the QLU-C10D functional domains of physical functioning (*p value* = *0.004*), role functioning (*p value* =  < *0.001*) and social functioning (*p value* =  < *0.001*), see Figure S10. A similar trend was observed with the symptom domains where more respondents undergoing surveillance because of symptoms reported more trouble with appetite (*p value* = *0.002*) and bowel function (*p value* = *0.01*) compared to those undergoing colonoscopy for surveillance and positive FOBT (Figure S10).

### Discriminant validity of EQ-5D-5L and QLU-C10D—Multivariable analysis

Following the univariate analysis, HRQoL at baseline and follow-up was significantly correlated with marital status, having a disability, fulltime employment, having private health insurance, a history of cancer, and a history of surgery (see Table S3). These variables were then adjusted for in the regression analysis. After controlling for these potential confounders, both EQ-5D-5L and QLU-C10D did not discriminate between colonoscopy findings at both baseline and follow-up (Table S4). EQ-5D-5L discriminated between respondents presenting with symptoms and positive FOBT or surveillance at baseline (*p value* < *0.05*), presenting with symptoms was associated with a lower HRQoL. QLU-C10D discriminated between participants presenting with symptoms and positive FOBT or surveillance colonoscopy. Participants undergoing colonoscopy due to symptoms had lower HRQoL compared to those undergoing a surveillance colonoscopy or due to positive FOBT at baseline (*p value* = *0.001*) and follow-up (*p value* = *0.006*).

## Discussion

This research aimed to assess HRQoL for individuals undergoing diagnostic or surveillance colonoscopy for CRC, using both the generic EQ-5D-5L and cancer-specific QLU-C10D and evaluate whether HRQoL differed based on the scale used. This study showed no differences in HRQoL between baseline and follow-up, using either scale, in patients undergoing screening, surveillance or symptom-driven colonoscopies, except at the dimension level. There is a paucity of studies assessing HRQoL in patients undergoing colonoscopy, however, we hypothesised that there would be differences based on colonoscopy findings. This study showed no differences in colonoscopy findings/outcomes, including for patients diagnosed with cancer with both EQ-5D-5L and QLU-C10D. This suggests a single scale of quality of life, would be sufficient to measure HRQoL in post-colonoscopy cohorts during future studies. However, it is also possible that the expected change was not detected due to the small sample size, resulting from premature study termination during the COVID-19 pandemic. Besides, given QLU-C10D is a new instrument, there is no prior information on the expected change in scores in our target patient population.

Symptomatic patients had a lower overall HRQoL, compared to patients undergoing colonoscopy for screening or surveillance purposes. Using QLU-C10D, after controlling for potential confounders, gastrointestinal symptoms were associated with lower HRQoL, compared to surveillance or positive FOBT. At the dimension level, more symptomatic individuals reported lower HRQoL at baseline (both measures) and at follow-up except for anxiety/depression where more under surveillance reported lower levels. The lower HRQoL reports, at both baseline and follow-up, in the group presenting with symptoms can be attributed to more patients being diagnosed with cancer in this group compared to those undergoing a surveillance colonoscopy or following a positive FOBT. However, the difference in colonoscopy findings (no polyps, polyps or cancer) between groups was not statistically significant. Also, the multivariate analysis controlled for colonoscopy findings as a confounder yet the HRQoL difference was still observed (see Table 1 and Table S3).

Our study is different from previous studies assessing HRQoL before and after colonoscopy [18, 43] because the respondents knew their colonoscopy results before the baseline assessment. Our results showed no association between colonoscopy findings and overall HRQoL. When assessing HRQoL (SF-36) and psychological distress among participants referred for colonoscopy following a positive FOBT, Vermeer et al. showed an increase in psychological dysfunction and worry following a cancer finding (2 weeks after colonoscopy) and a decline for those with no cancer. For those with a cancer diagnosis, psychological dysfunction declined to pre-colonoscopy measurements after 6 months [44]. Considering that participants in our study, unlike the above study, knew their colonoscopy findings at baseline (38 days after colonoscopy), this was not a true reflection of their baseline. This means that the change happened before the HRQoL assessment, and the baseline value reflected the post-colonoscopy HRQoL, which suggests that participants are returning to their true baseline level earlier than 6 months. More individuals undergoing surveillance colonoscopy reported problems with anxiety or depression (EQ-5D-5L) at baseline compared to those due to symptoms or positive FOBT, but this was not observed with emotional functioning on the QLU-C10D or after controlling for potential confounders. These results with the EQ-5D-5L agree with studies that suggest that individuals taking part in routine screening/surveillance report higher levels of anxiety over the possibility of cancer [45, 46]. Yet the lack of difference in emotional functioning observed with QLU-C10D is also supported by several studies that argue that participation in colorectal cancer screening [16] or the results of a colorectal cancer screening colonoscopy have no effects on participants' psychological well-being [17, 47].

After controlling for potential confounders, participants undergoing colonoscopy because of symptoms had lower HRQoL/utility scores with QLU-C10D at both baseline and one year compared to those having surveillance colonoscopy or because of a positive FOBT (and only at baseline with EQ-5D-5L). At the dimension level, more individuals presenting with symptoms reported lower levels for usual activities and pain/discomfort on the EQ-5D-5L at baseline. Using QLU-C10D, participants with symptoms reported lower levels for role function, appetite and bowel problems at both baseline and follow-up. This finding, particularly appetite and bowel problems is not surprising because these are common presenting signs in people undergoing colonoscopy in general [48] and under investigation for colorectal cancer [49]. It is particularly important to note that this difference is observed with the cancer-specific scale, QLU-C10D, whose dimensions include disease-related symptom dimensions, unlike the generic scale.

We, therefore, explored whether the scales used were suitable and sensitive to HRQoL changes in this population. Participants reporting full health with EQ-5D-5L (the best level on all dimensions) still had problems according to QLU-C10D, particularly with fatigue, sleep and role function. This suggests that QLU-C10D is more sensitive than the generic scale in picking up differences in HRQoL and could potentially be used in all future studies of post-colonoscopy HRQoL, including in non-cancer cohorts. This result was similar to that observed when EQ-5D-5L was compared to the cancer-specific HRQoL scale FACT-8D [50] and when the three-level version of EQ-5D, EQ-5D-3L was compared to the cancer-specific EORTC-8D, which like QLU-C10D, is derived from the EORTC QLQ-C30 [19]. Both studies showed that compared to the cancer-specific scales, the generic scales failed to detect impairments with fatigue and sleep disturbances. These findings highlight the gap within the EQ-5D descriptive system, supporting the argument by Chen and Olsen, and Sprouk et al., 2021 to add sleep and fatigue bolt-on dimensions to the EQ-5D descriptive system [51, 52].

Similar to other studies [19, 21], our results suggest that when assessing short-term HRQoL outcomes in populations undergoing diagnostic colonoscopy for cancer, the cancer-specific scale is more sensitive compared to generic scales of HRQoL. The sensitivity of a scale is critical when assessing the cost-effectiveness of interventions in a particular population. A more sensitive scale will detect change where change would otherwise not have been detected with a less sensitive scale and this influences the incremental cost-effectiveness ratio (ICER) results, and subsequently, the cost-effectiveness decision. While decision-making bodies (e.g. NICE, PBAC and MSAC) require the use of generic scales for purposes of economic evaluations [53–55], we propose that economic evaluations assessing HRQoL in this setting should consider both the generic scale and the cancer-specific scale as it is more sensitive to differences.

Limitations of this study include the low response rate (38%), which although similar to other quality-of-life postal surveys [56], can be attributed to the premature termination of the study data collection due to the COVID-19 pandemic in 2019/2020. We also observed that more participants undergoing colonoscopy due to symptoms and those diagnosed with cancer did not respond to the survey, which indicates a response bias. There are known differences based on diagnosis after colonoscopy and that was hypothesised in this study; however, the study did not have the power to detect the expected change due to the small sample size, resulting from premature study termination. Furthermore, given QLU-C10D is a new instrument, there is no prior information on the expected change in scores in our target patient population. We therefore recommend a larger future study with both asymptomatic and symptomatic patients. Future research should assess anxiety levels and cancer concerns in symptomatic patient cohorts, to assess whether these concerns persist despite a colonoscopy ruling out cancer, and if so, how such concerns can be better allayed. Also, our baseline survey was conducted after participants had received their colonoscopy results. As such we cannot provide a direct comparison to previous studies whose baseline assessments were before the colonoscopy procedure. Another possible limitation is that the EQ-5D-5L utility scores were generated based on a UK population value set as recommended by NICE [31], while the QLU-C10D was valued based on an Australian population. Another possible limitation is the ordering effect as the survey maintained the generic EQ-5D-5L before the EORTC-QLQ-C30 at both baseline and follow-up. However, studies have shown that presentation order only has a marginal effect on the patient responses to HRQoL scales [57, 58].

## Conclusion

HRQoL does not change one year following a diagnosis of the bowel at colonoscopy and it does not differentiate between different colonoscopy diagnoses including cancer. However, patients undergoing colonoscopy because of symptoms have poorer HRQoL compared to those undergoing surveillance colonoscopy for cancer. In addition, a cancer-specific scale is more sensitive than a generic scale to HRQoL differences in patients undergoing colonoscopy.

### Supplementary Information

Below is the link to the electronic supplementary material.

Supplementary file1 (DOCX 763 KB)

## Data Availability

The datasets generated during and/or analysed during the current study are available from the corresponding author upon reasonable request.
