# Supplementary material for: Health utility assessments in individuals undergoing diagnostic and surveillance colonoscopy: improved discrimination with a cancer-specific scale
Source: Cancer Causes Control. 2023 Sep 25;35(2):347–57. doi: 10.1007/s10552-023-01789-6 (PMC10787680; doi:10.1007/s10552-023-01789-6)
Supplement: Supplementary file 1 — Supplementary file1 (DOCX 763 KB) [file 10552_2023_1789_MOESM1_ESM.docx]

**Supplementary data**

**Title: Health Related Quality of Life in individuals undergoing diagnostic and surveillance colonoscopy: improved discrimination with a cancer specific questionnaire**

## Figure S1: Flow chart for enrolment in the study

644 scheduled for colonoscopy and sent the survey

**Excluded:**

385 did not return the survey

5 had a pre-existing bowel condition

**Colorectal cancer**

N=44

**No polyps removed at colonoscopy**

N=75

**Polyp removed at colonoscopy**

N=128

N=91

N=57

**Early stage**

(Stage I and II)

N=22

**Advanced stage (**Stage III and IV)

N=22

N=12

N=11

*Completion of follow-up survey 12 months later*

Symptoms (n=54), Positive FOBT (n=65), Surveillance (n=57)

254 eligible for analysis

Symptoms (n=87), Positive FOBT (n=92), Surveillance (n=67)

**Table S1: Demographic characteristics for responders and non-responders at follow-up**

| **Variable** | **Responders (N=176)** | **Non-responders**  **(n= 69)** | **p-value^1^** |
| --- | --- | --- | --- |
| Male | 95 (54%) | 39 (57%) | 0.877 |
| Low socioeconomic status | 71 (41%) | 36 (52%) | 0.139 |
| Married | 108 (61%) | 45 (65%) | 0.858 |
| Higher Education | 83 (47%) | 32 (46%) | 0.925 |
| Full Time Worker | 33 (19%) | 9 (13%) | ***0.032*** |
| Retired | 83 (33.3%) | 32 (46%) | 0.618 |
| Have private health insurance | 74 (43%) | 23 (33%) | 0.139 |
| Have a disability | 37 (21%) | 19 (28%) | 0.244 |
| Had surgery in the last 12 months | 43 (26%) | 16 (24%) | 0.436 |
| Previous or current cancer | 45 (26%) | 29 (43%) | ***0.000*** |
| Age [mean (SD)]^2^ | 65.2 (7.7) | 65.1 (9.1) | 0.335 |
| **Age category** |  |  |  |
| <55 | 13 (7%) | 11 (16%) | 0.181 |
| 55-65 | 68 (39%) | 19 (28%) |  |
| 66-75 | 84 (48%) | 30 (45%) |  |
| >75 | 9 (5%) | 7 (11%) |  |
| **Indication for colonoscopy^3^** |  |  |  |
| Surveillance | 54 (32%) | 12 (17%) | *0.014* |
| Positive FOBT | 65 (37%) | 25 (36%) |  |
| Symptoms | 57 (31%) | 32 (46%) |  |
| **Colonoscopy findings^3^** |  |  |  |
| No polyp | 57 (32%) | 13 (20%) | *0.003* |
| Polyp | 91 (52%) | 34 (52%) |  |
| Cancer | 24 (14%) | 19 (29%) |  |
| stage I and stage II | 13 | 9 |  |
| stage III and stage IV | 11 | 10 |  |

*1=p-value using chi square test; 2=p-value using Wilcoxon ranksum test; 3=p-value using k-wallis test*

## Figure S2: Ceiling effects of EQ-5D-5L dimensions at baseline and follow-up

*For the utility score = percentage reporting full health*

## Figure S3: Ceiling effects of QLU-C10D dimensions at baseline and follow-up

*PF=Physical function, RF=Role function, SF=Social function, EF=Emotional function, PA=Pain, FA=Fatigue, SL=Sleep, AP=Appetite, NA=Nausea, BP=Bowel problems*

**Figure S4: Floor effects of EQ-5D-5L dimensions at baseline and follow-up**

**Figure S5: Floor effects of QLU-C10D dimensions at baseline and follow-up**

## Table S2: Distribution (%) of QLU-C10D for participants with full health on EQ-5D-5L but not on QLU-C10D*

| **Level** | **Physical function** | **Role function** | **Social function** | **Emotional function** | **Pain** | **Fatigue** | **Sleep** | **Appetite** | **Nausea** | **Bowel problems** |
| --- | --- | --- | --- | --- | --- | --- | --- | --- | --- | --- |
| **Baseline n=51** | |  |  |  |  |  |  |  |  |  |
| 1 (best level) | 73 | 92 | 90 | 86 | 86 | 31 | 39 | 90 | 96 | 67 |
| 2 | 23 | 8 | 6 | 14 | 14 | **61** | **59** | 10 | 4 | 25 |
| 3 | 0 | 0 | 2 | 0 | 0 | 8 | 2 | 0 | 0 | 6 |
| 4 (worst level) | 4 | 0 | 2 | 0 | 0 | 0 | 0 | 0 | 0 | 2 |
| **Follow-up n=29** | |  |  |  |  |  |  |  |  |  |
| 1 (best level) | 83 | 39 | 90 | 93 | 69 | 28 | 55 | 90 | 93 | 59 |
| 2 | 14 | **59** | 7 | 7 | 31 | **69** | 38 | 10 | 4 | 41 |
| 3 | 0 | 2 | 0 | 0 | 0 | 3 | 4 | 0 | 4 | 0 |
| 4 (worst level) | 3 | 0 | 3 | 0 | 0 | 0 | 4 | 0 | 0 | 0 |

**Percentages rounded off to the nearest whole number; Bold= where more than 50% reported less than the best level*

#### Figure S6: Responses to dimensions of the EQ-5D-5L at baseline – differences between indications for colonoscopy


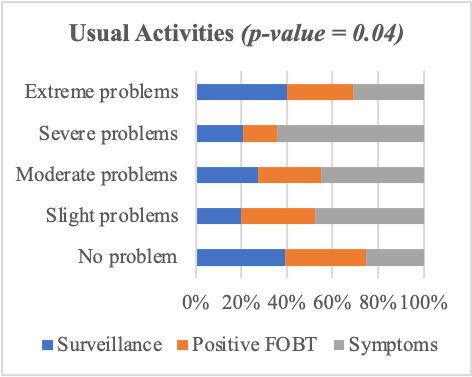

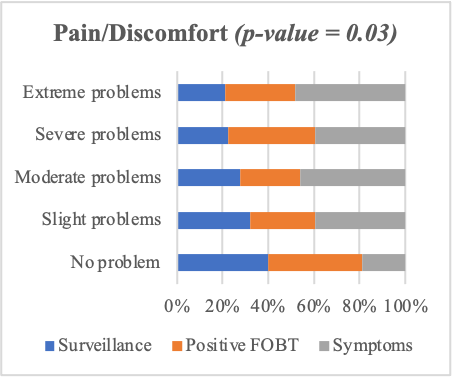

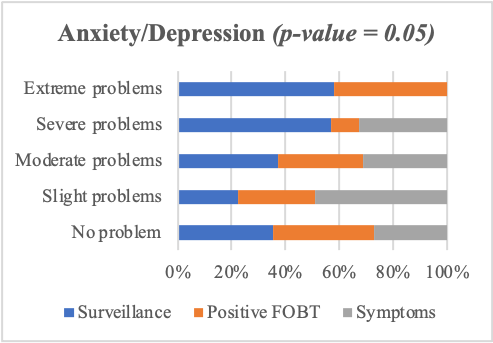


#### Figure S7: Responses to dimensions of the QLU-C10D at baseline – differences between colonoscopy findings


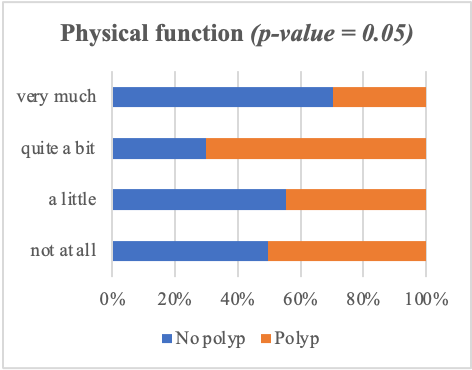

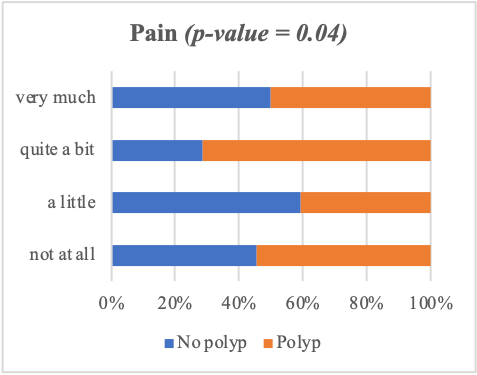

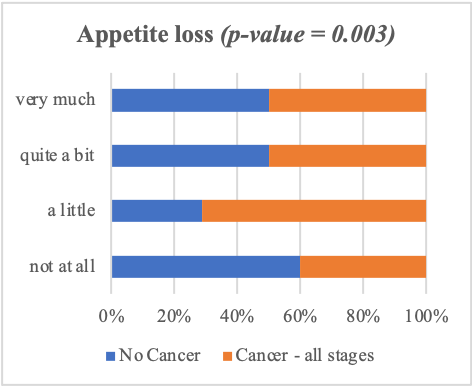

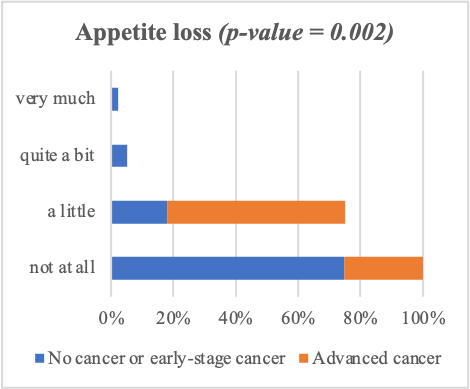


#### Figure S8: Responses to dimensions of the QLU-C10D at baseline – significant differences between indications for colonoscopy


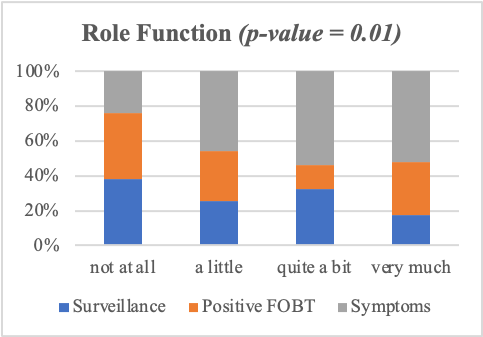

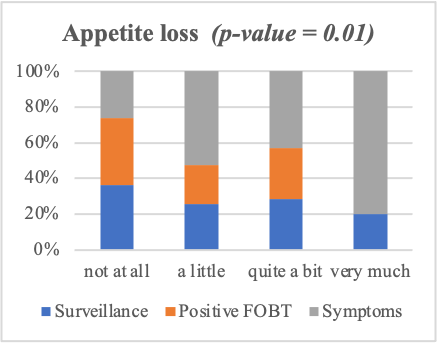

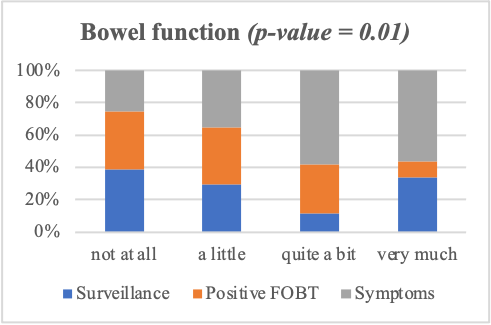


#### Figure S9: Responses to dimensions of the QLU-C10D at follow-up – significant differences between colonoscopy findings


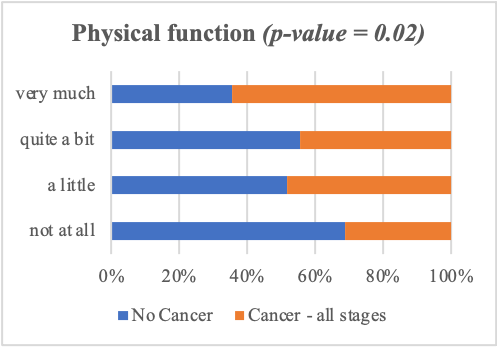

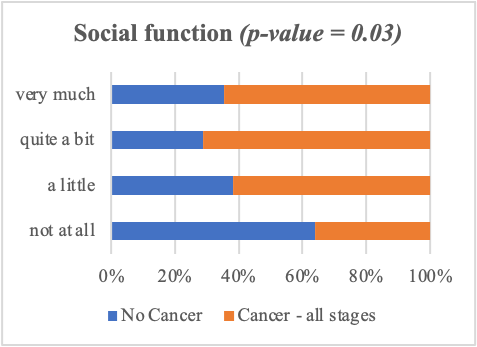

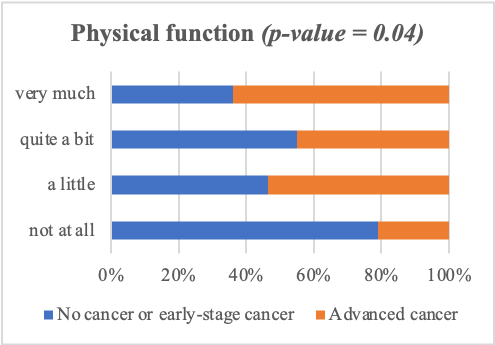

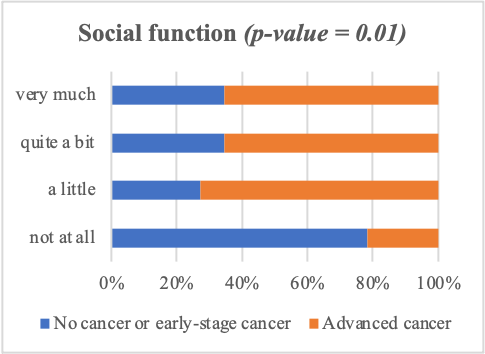

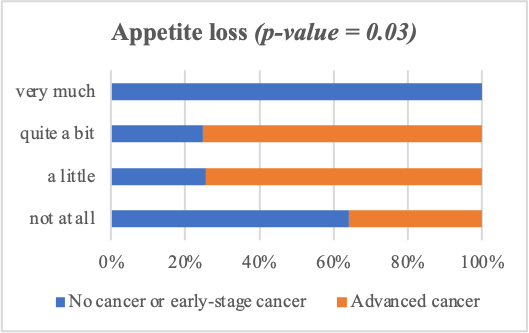


#### Figure S10: Responses to dimensions of the QLU-C10D at follow-up – significant differences between indications for colonoscopy


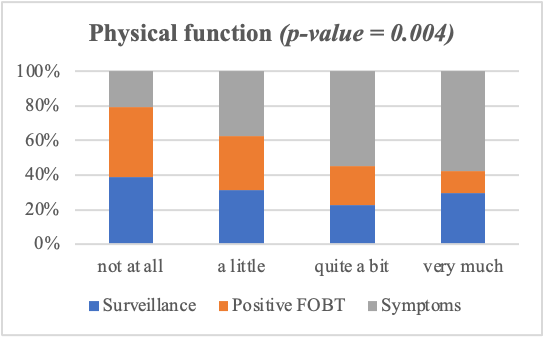

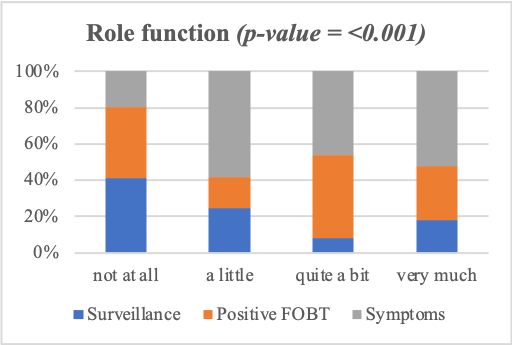

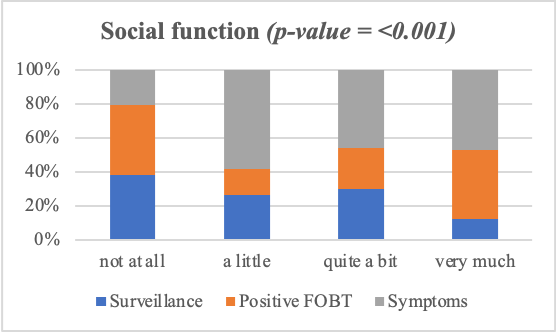

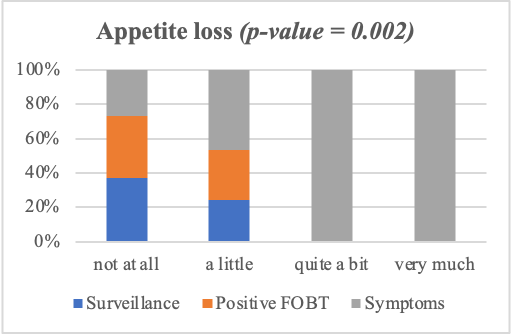

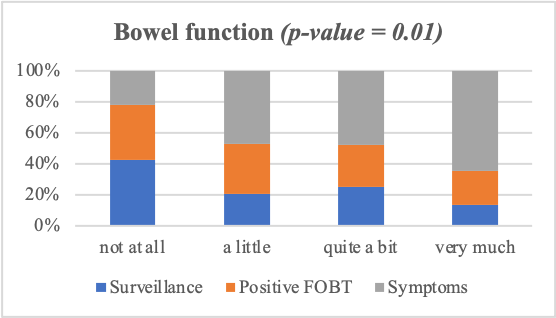


## Table S3: Univariate analysis

| **Variable** | **Baseline** | | | | **Follow-up** | | | |
| --- | --- | --- | --- | --- | --- | --- | --- | --- |
|  | **EQ5D-5L** | **p-value** | **QLU-C10D** | **p-value** | **EQ5D-5L** | **p-value** | **QLU-C10D** | **p-value** |
| Sex | 0.001 | 0.991 | -0.065 | 0.310 | -0.113 | 0.135 | -0.146 | 0.054 |
| Age | -0.042 | 0.517 | -0.105 | 0.105 | -0.018 | 0.815 | 0.036 | 0.634 |
| Comorbidities | -0.088 | 0.179 | -0.110 | 0.091 | -0.102 | 0.210 | -0.118 | 0.133 |
| History of surgery | -0.217 | ***0.001*** | -0.261 | ***0.000*** | -0.138 | 0.074 | -0.230 | ***0.003*** |
| History of cancer | -0.191 | ***0.003*** | -0.249 | ***0.000*** | -0.179 | ***0.019*** | -0.191 | ***0.012*** |
| Having a disability | -0.593 | ***0.000*** | -0.564 | ***0.000*** | -0.499 | ***0.000*** | -0.510 | ***0.000*** |
| Marital status | 0.046 | 0.473 | 0.072 | 0.263 | 0.122 | 0.107 | 0.191 | ***0.011*** |
| Being retired | -0.058 | 0.364 | -0.093 | 0.144 | 0.020 | 0.788 | 0.078 | 0.304 |
| Fulltime employment | 0.142 | ***0.026*** | 0.173 | ***0.006*** | 0.098 | 0.197 | 0.025 | 0.743 |
| Higher education | 0.111 | 0.082 | 0.087 | 0.174 | 0.104 | 0.169 | 0.067 | 0.378 |
| Private health insurance | 0.241 | ***0.000*** | 0.278 | ***0.000*** | 0.155 | ***0.042*** | 0.202 | ***0.008*** |

## Table S4: Discriminant validity between main colonoscopy diagnostic groups and indications after controlling for potential confounders^1^

| **Variable** | **Baseline** | | | | **Follow-up** | | | |
| --- | --- | --- | --- | --- | --- | --- | --- | --- |
|  | **EQ-5D-5L** | | **QLU-C10D** | | **EQ-5D-5L** | | **QLU-C10D** | |
|  | **Coefficient** | **SE** | **Coefficient** | **SE** | **Coefficient** | **SE** | **Coefficient** | **SE** |
| **Colonoscopy finding** |  |  |  |  |  |  |  |  |
| Cancer^3^ vs Non-Cancer^2^ | 0.032 | 0.053 | -0.032 | 0.038 | 0.033 | 0.079 | 0.061 | 0.057 |
| Advanced Stage Cancer^5^ vs Non-Cancer & Early-Stage Cancer^4^ | 0.036 | 0.068 | 0.079 | 0.050 | 0.029 | 0.094 | -0.005 | 0.070 |
| **Indication for colonoscopy** |  |  |  |  |  |  |  |  |
| Positive FOBT vs Surveillance | 0.029 | 0.036 | -0.015 | 0.025 | 0.013 | 0.041 | 0.026 | 0.030 |
| Symptoms vs Surveillance | ***-0.076**** | ***0.037*** | ***-0.080**** | ***0.027*** | -0.061 | 0.045 | ***-0.115**** | ***0.035*** |
| Symptoms vs Positive FOBT | ***-0.105**** | ***0.034*** | ***-0.095**** | ***0.024*** | -0.074 | 0.043 | ***-0.089*** | ***0.032*** |
| N | 231 |  | 231 |  | 161 |  | 161 |  |

**p-values =<0.05; ^1^Confounders controlled for were marital status, fulltime employment, having private health insurance, having a disability, previous history of surgery and cancer, and the indication for undergoing colonoscopy; ^2^Non-cancer = normal, non-neoplastic and polyp; ^3^Cancer = all stages of cancer; ^4^Early stage cancer = cancer stage I & II; ^5^Advanced cancer stage = cancer stage III & IV*; *FOBT=Faecal Occult Blood Test*
